# Review of recent developments in capacitor-type heat flow switching devices

**DOI:** 10.1080/14686996.2025.2590797

**Published:** 2025-11-24

**Authors:** Keisuke Hirata, Tsunehiro Takeuchi

**Affiliations:** a Graduate School of Engineering, Toyota Technological Institute, Nagoya, Japan; b Research Center for Smart Energy Technology of Toyota Technological Institute, Nagoya, Japan

**Keywords:** Thermal conductivity, lattice thermal conductivity, electron thermal conductivity, heat flow switching, capacitor, silver chalcogenides

## Abstract

This review summarizes our recent developments in capacitor-type heat flow switching devices that enable active control of heat flow magnitude through the modulation of electron thermal conductivity. We initially demonstrated the feasibility of a capacitor-type heat flow switching device using silver chalcogenides, Ag_2_S_1–*x*_Se_*x*_, as an electrode material with very low lattice thermal conductivity (≤0.5 W m^−1^ K^−1^). We achieved significant enhancements in heat flow switching performance through subsequent improvements, including electrode thinning and the implementation of an electric double-layer capacitor structure with ionic liquids. The switching ratio improved from an initial value of 1.1 at the bias voltage of *V*_B_ = +3 V to 1.9 at *V*_B_ = +2.4 V, while response times were estimated to be less than 0.2 s. This review discusses the operating principles, experimental methods, and performance metrics across different device configurations, highlighting the critical role of electrode materials with extremely low lattice thermal conductivity. Our findings establish a promising candidate for practical thermal management applications that require rapid and reliable heat flow control without mechanical components.

## IMPACT STATEMENT

This review summarizes developments in capacitor-type heat flow switching devices enabling active thermal management via electron thermal conductivity modulation, achieving a switching ratio of 1.9 with sub-second response times without mechanical components.

## GRAPHICAL ABSTRACT

**Figure:**
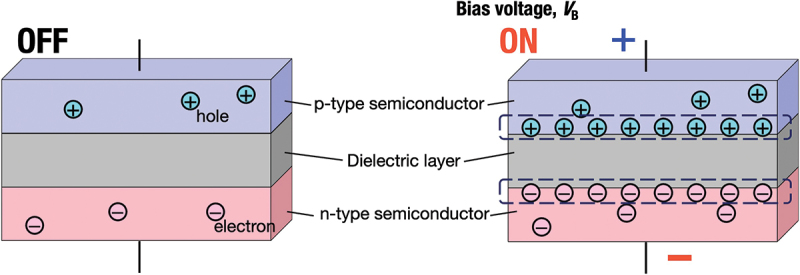

## Introduction

Thermal management has emerged as a critical field for building energy-efficient systems and for utilizing waste heat. Recent reports indicate that roughly 60 – 70% of primary energy is dissipated as unused thermal energy [1,2]. The effective utilization of this waste heat and precise control of thermal energy flow represents significant opportunities for improving energy efficiency across various technological domains. Heat flow switching – the ability to modulate heat transfer on demand – constitutes a key technology for advanced thermal management systems with applications spanning energy harvesting, thermal computing, electronic device cooling, and thermal energy storage [3,4].

In recent years, non-mechanical approaches have been explored extensively, each with distinct advantages and limitations [3,4]. Heat conduction in solid materials is governed by Fourier’s law: $J_{Q}=-\kappa \nabla T$, where **J**_*Q*_ represents heat flow density, ∇*T* is the temperature gradient, and *κ* denotes thermal conductivity. Under a fixed temperature gradient, a variation in the magnitude of heat flow requires a significant change in the thermal conductivity. In typical solid materials possessing electrical conduction, thermal conductivity comprises contributions from electrons and lattice vibrations (phonons):

$$\kappa =\kappa_{\mathrm{ele}}+\kappa_{\mathrm{lat}}\ldots$$

where *κ*_ele_ and *κ*_lat_ represent electron and lattice thermal conductivity, respectively. Additional contributions from other collective excitations such as magnons (*κ*_mag_), spinons (*κ*_spin_), or polarons (*κ*_pol_) may exist in specific materials. Developing effective heat flow switching devices necessitates identifying materials and mechanisms that allow us to significantly modulate one or more of these contributors while maintaining that other contributions are kept negligibly small.

It would be natural to consider that a significant variation in thermal conductivity is realized at the structure phase transition, especially at solid-liquid and order-disorder phase transitions. Such a phase transition can achieve a heat-flow switching ratio of 2 – 10 times [5–11]. Here, we mainly consider the above room temperature techniques. For example, VO_2_-based materials undergo substantial changes (~2 times) in thermal conductivity across phase transitions, but their operation is limited to narrow temperature ranges near the transition temperature (typically 240 – 340 K for V_*x*_W_1–*x*_O_2_ (*x* = 0, 0.021, 0.026, and 0.045) [6]). Electrochemical approaches employ redox reactions to modify material composition and thermal properties. While exhibiting switching ratios up to 4 – 10 times, these systems suffer from relatively slow response times (minutes) and might degrade over multiple operational cycles [12–15]. Magnetically controlled heat flow switching devices utilize magnetic fields to alter thermal conductivity through spin-dependent electron scattering [16–19]. Although they offer non-contact actuation, they require constant power to maintain the switched state and often exhibit strong temperature-dependent performance.

The capacitor-type heat flow switching devices developed in our research offer a fundamentally different approach that overcomes these limitations. By modulating carrier concentration through applied bias voltage, these devices provide non restricted operation by a phase-transition temperature, rapid response times (sub-second), absence of mechanical moving parts, compatibility with semiconductor processing techniques, low operating voltages, and potential for long-term reliability and cyclability [20,21].

This review aims to comprehensively document the development and progressive improvement of capacitor-type heat flow switching devices, highlighting the fundamental operating principles and theoretical considerations that underpin the technology. We trace the evolution from initial proof-of-concept to optimized devices across three generations, analyzing key materials innovations and their impact on device performance. A comparative analysis with other thermal switching technologies is presented to contextualize our achievements, followed by discussion of future research directions and potential applications. Through this comprehensive review, we provide a foundation for further improvement toward practical thermal management applications.

**Figure 1.**
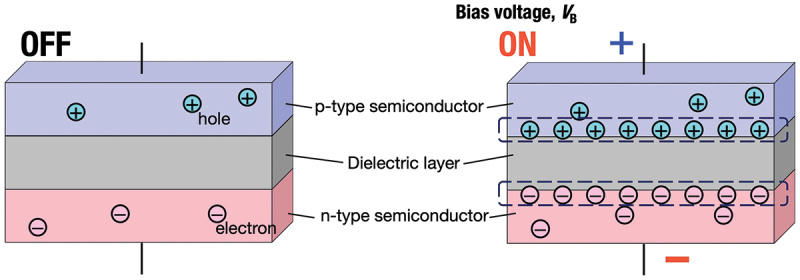
Schematic illustration of the principle of capacitor-type heat flow switching device. When a bias voltage is applied, carrier concentration increases around the semiconductor-dielectric interfaces, enabling control of electron thermal conductivity in semiconductor electrodes through carrier density modulation. The resulting thermal conductivity variations can be detected in both cross-plane and in-plane directions by selecting appropriate measurement techniques.

Figure 1 illustrates the fundamental operating principle of our capacitor-type heat flow switching devices. The device consists of a layered structure with p-type and n-type semiconductor electrodes separated by a dielectric layer. When a forward bias voltage is applied, carriers (electrons and holes) are introduced near the electrode-dielectric interfaces, increasing carrier concentration in these regions. This increase in carrier concentration enhances electrical conductivity and, consequently, electron thermal conductivity according to the Wiedemann-Franz law [22,23]: *κ*_ele_ = *L*_0_*σT* where *κ*_ele_, *L*_0_, *σ*, and *T* represent electron thermal conductivity, the Lorenz number (2.44 × 10^−8^ W Ω K^−2^), electrical conductivity, and temperature, respectively. Thus, by modulating electrical conductivity through carrier concentration changes, the electron thermal conductivity can be actively controlled.

The performance of capacitor-type thermal switching elements strongly depends on the physical properties of the employed materials. The thermal conductivity of typical semiconductors can be represented as the sum of electron thermal conductivity and lattice thermal conductivity. Therefore, the resulting heat flow switching ratio (*SR*) can be expressed by the following equation:

$$\mathrm{SR}=\frac{\kappa_{\mathrm{total}}\left(V_{B}\right)}{\kappa_{\mathrm{total}}\left(0\right)}=\frac{\kappa_{\mathrm{ele}}\left(V_{B}\right)+\kappa_{\mathrm{lat}}}{\kappa_{\mathrm{ele}}\left(0\right)+\kappa_{\mathrm{lat}}}$$

Maximizing the change in electron thermal conductivity requires materials with appropriate semiconductor transport properties that enable significant carrier concentration modulation upon bias voltage application. The above equation also reveals that maximizing the switching ratio requires minimizing lattice thermal conductivity, since lattice thermal conductivity remains constant regardless of bias voltage and represents a non-modulatable component that diminishes the overall switching effect. Materials with extremely low *κ*_lat_ are therefore essential for high-performance capacitor-type heat flow switching devices. For a material with *κ*_lat_ = 10 W m^−1^ K^−1^ and initial electrical conductivity *σ*(0) = 1 S cm^−1^ at *T* = 300 K, the initial electron thermal conductivity is *κ*_ele_(0) ~ 0.007 W m^−1^ K^−1^ according to the Wiedemann-Franz law. If carrier accumulation increases the electrical conductivity by a factor of 1000 to *σ*(*V*_B_) = 1000 S cm^−1^, then *κ*_ele_(*V*_B_) ~ 7 W m^−1^ K^−1^, resulting in a switching ratio of *SR* = (7 + 10) / (0.007 + 10) ~ 1.7. On the other hand, if *κ*_lat_ could be reduced to 1 W m^−1^ K^−1^ while maintaining the same electrical conductivity changes, the switching ratio would increase to *SR* ~7.9, highlighting the critical importance of minimizing lattice thermal conductivity for achieving high switching performance.

Furthermore, optimizing device geometry (thin electrode layers) becomes critical since carrier accumulation occurs primarily within a few nanometers of the electrode-dielectric interface. The accumulated charge density at the electrode-dielectric interface can be expressed as *Q* = *εV*_B_ / *d* where *Q*, *ε*, *V*_B_, and *d* represent the stored charge, dielectric constant of the insulating layer, applied bias voltage, and the thickness of the dielectric layer, respectively [24]. This accumulated charge leads to a carrier concentration change (Δ*n*) within a characteristic screening length near the interface: Δ*n* = *Q* / (|*e*| *S t*) where *S, t*, and *e* denote the electrode area, the effective thickness of the carrier accumulation layer, and the elementary charge, respectively. These considerations guided our material selection and device design across the different generations of heat flow switching devices, as will be detailed in subsequent sections.

## Development of capacitor-type heat flow switching devices

### Initial proof-of-concept experiments using silver chalcogenides as electrodes

Our research on capacitor-type heat flow switching devices began with proof-of-concept experiments designed to validate the fundamental operating principle [20]. This initial study focused on demonstrating that carrier concentration modulation via bias voltage could indeed produce measurable changes in thermal conductivity. For the first-generation device, we selected *x* = 0.3 and 0.4 in the Ag_2_S_1–*x*_Se_*x*_ system as the electrode material based on their exceptionally low lattice thermal conductivity (0.4 – 0.5 W m^−1^ K^−1^) due to strong anharmonic lattice vibrations and moderate semiconducting electrical conductivity (10 – 50 S cm^−1^) [20,25].

Silver chalcogenides Ag_2_S_0.7_Se_0.3_ and Ag_2_S_0.6_Se_0.4_ were synthesized via self-propagating high-temperature synthesis [26] (SHS) and melting methods, followed by hot pressing to produce dense polycrystalline bulks. Utilizing the ductile nature of the Ag_2_S-type crystal structure observed in these compositions [25,27–29], the obtained bulks were mechanically rolled into ribbon-shaped samples approximately ~10 μm in thickness. To construct a capacitor-type device, a silver chalcogenide ribbon served as one electrode, with amorphous Si (500 nm in thickness) deposited as the dielectric layer via RF magnetron sputtering. Mo (500 nm in thickness) was employed as the counter electrode, deposited using the same RF magnetron sputtering.

**Figure 2.**
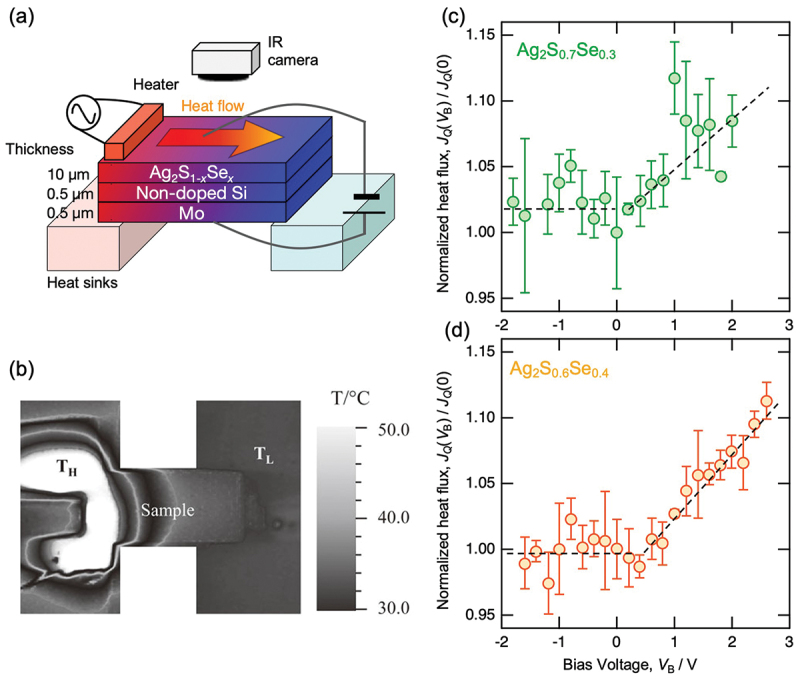
(a) Schematic illustration of the first capacitor-type heat flow switching device consisting of Ag_2_S_1–*x*_Se_*x*_ (*x* = 0.3 and 0.4) / amorphous Si / Mo under AC heating measurement with and without bias voltage. (b) Temperature distribution at the surface of silver chalcogenide electrode observed using an IR camera. (c, d) Bias voltage dependence of normalized heat flow observed using the capacitor-type heat flow switching device shown in (a). These figures are reconstructed from the data of Ref. [20].

To verify the fabricated capacitor-type heat flow switching device, we investigated the bias voltage dependence of thermal conductivity by applying various bias voltages between the Mo layer and silver chalcogenide electrodes, using the AC heating method [30]. A schematic diagram of the experimental setup is shown in Figure 2(a). An AC temperature wave was generated using a resistance heater, and the spatial-temporal temperature distribution on the surface of the silver chalcogenides electrode was captured by an infrared camera, as shown in Figure 2(b). Thermal diffusivity was calculated by measuring the phase difference and amplitude ratio of temperature waves at multiple points on the sample, thereby determining the bias voltage dependence of heat flow.

Figure 2(c,d) show the bias voltage dependence of heat flow obtained in the devices using Ag_2_S_0.7_Se_0.3_ and Ag_2_S_0.6_Se_0.4_ as the electrodes, respectively. In both devices, as the forward bias voltage increased, the heat flow increased linearly, confirming their functionality as capacitor-type heat flow switching devices. In the device using Ag_2_S_0.6_Se_0.4_, an increase in heat flow of ~10% was observed when a forward bias voltage of 3 V was applied compared to the zero-voltage condition. Although this change was indeed small, we succeeded in demonstrating the validity of the operating principle of the capacitor-type heat flow switching device.

The main factor limiting the obtained switching ratio (1.1 times) was most likely due to the relatively large thickness (~10 μm) of the Ag_2_S_0.6_Se_0.4_ electrode. Because carrier accumulation occurs only within a few nanometers of the interface, only about 0.1% of the electrode volume was affected by the carrier modulation. Additionally, the applied bias voltage was limited due to the performance constraints of the experimental setup used in this study.

Regarding the bias voltage dependence of heat flow, an increase in thermal conductivity was observed under forward bias voltage, while almost no change was observed under reverse bias voltage. This asymmetry can be attributed to the n-type semiconducting nature of the silver chalcogenide electrode. Under reverse bias voltage application, carriers were depleted near the interface between the silver chalcogenide and the electrode used to apply the bias voltage, forming an insulator-like state, known as a depletion layer, to prevent the bias voltage from reaching the interface between Ag_2_S_1–*x*_Se_*x*_ electrode and amorphous Si. Although the obtained heat flow switching ratio was not sufficiently large, this experimental demonstration confirmed the feasibility of capacitor-type heat flow switching devices and clarified essential design guidelines for performance improvement, such as reducing the thickness of the electrode material.

### Thin-film implementation using amorphous Si-Ge

Based on the insights gained from our first experiments, we developed a capacitor-type heat flow switching device using thin-film electrodes to improve the heat flow switching ratio by increasing the proportion of volume where carrier concentration modulation occurs [21].

**Figure 3.**
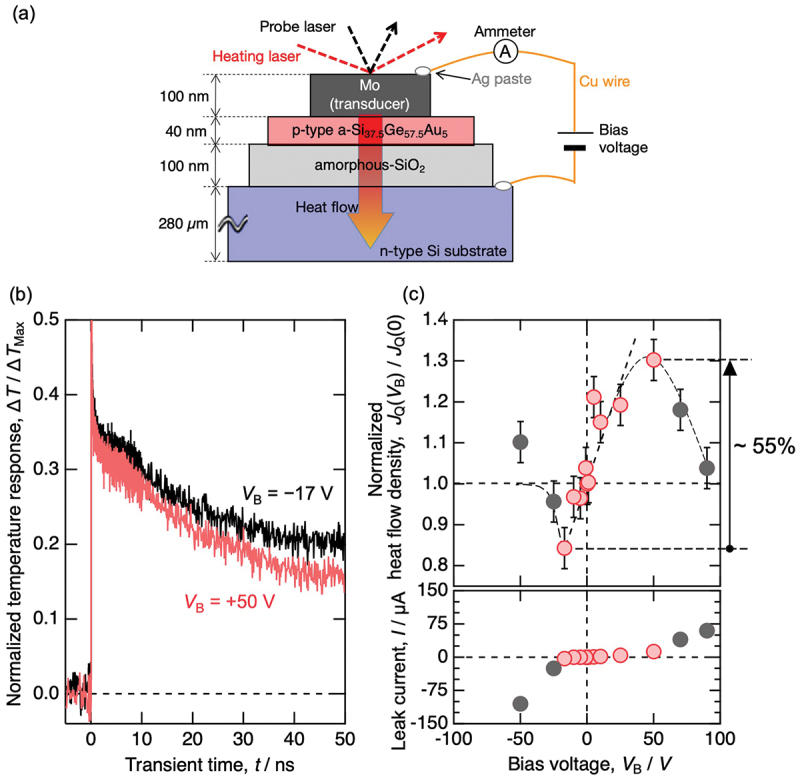
(a) Schematic illustration of thin film capacitor-type heat flow switching device and bias voltage-dependent heat flow measurement setup using time-domain thermoreflectance (TDTR) method. (b) Time-dependent normalized temperature response of the TDTR measurement with the bias voltage of *V*_B_ = +50 V and *V*_B_ = −17 V. (c) Bias voltage dependent heat flow and leakage current. These figures are reconstructed from the data of Ref. [21].

As shown in Figure 3(a), the second device consisted of the following components: an n-type Si substrate (280 μm thickness, electrical resistivity ~40 Ω cm [21]) as the bottom electrode, thermally oxidized SiO_2_ (100 nm in thickness) as the dielectric layer, an amorphous p-type Si_37.5_Ge_57.5_Au_5_ alloy (40 nm in thickness) as the top electrode, and Mo (100 nm in thickness) as both the electrode for bias voltage application and as a transducer layer for heat flow measurements using time-domain thermoreflectance (TDTR) method [31,32] which provides picosecond time resolution and enables layer-by-layer analysis of thermal conductivity at the nanometer scale. The device structure and heat flow evaluation methodology were improved over the first device. Specifically, the electrode thickness was reduced from 10 μm to 40 nm, and the experimental configuration was adjusted to enable cross-plane heat flow measurements aligned with the direction of carrier accumulation.

The fabrication process began with thermal oxidation of an n-type Si wafer in a mixed H_2_ and O_2_ gas flow to form the amorphous SiO_2_ layer. Subsequently, the p-type amorphous Si-Ge-Au layer was deposited on the amorphous SiO_2_ at room temperature using a molecular beam deposition (MBD) apparatus. Finally, a 100 nm thick Mo layer was deposited on the p-type amorphous Si-Ge-Au layer by RF magnetron sputtering. The composition of the Si-Ge-Au alloy used for the top electrode was systematically optimized to minimize lattice thermal conductivity while maintaining moderate electrical conductivity (1–100 S cm^−1^). According to the previous reports [33], the lowest lattice thermal conductivity (*κ*_lat_ ~ 0.7 W m^−1^ K^−1^) in amorphous Si-Ge thin films is observed near the Si_40_Ge_60_ composition. By introducing Au as a p-type dopant and evaluating Si_40–0.5*x*_Ge_60–0.5*x*_Au_*x*_ (*x* = 3, 5, 15, and 20 at.%), we selected *x* = 5 at.% (Si_37.5_Ge_57.5_Au_5_) as the optimal composition. This composition provided a low lattice thermal conductivity of ~0.7 W m^−1^ K^−1^, electrical conductivity of 8.2 S cm^−1^, and a fully amorphous structure without nanocrystalline precipitates that appeared at higher Au concentrations [21].

Figure 3(b) shows the time-dependent raw TDTR signals observed for the second heat flow switching device under bias voltages of *V*_B_ = +50 V and *V*_B_ = −17 V. At *t* = 0, the heating laser was irradiated, causing a temperature increase, followed by a cooling process. Faster decay indicates higher thermal conductivity. A clear difference in the raw TDTR signals between the forward and reverse bias voltages was observed, successfully confirming the heat flow change occurred throughout the entire device thickness.

In Figure 3(b), fitting analysis was conducted on the time-domain data (0.1–2 ns), which is most likely attributed exclusively to the Si-Ge-Au layer, in order to calculate heat flow. Figure 3(c) shows the bias voltage dependence of heat flow observed in the second heat flow switching device. The heat flow increased linearly with increasing bias voltage, showing a maximum increase of +55%. Assuming a similar linear bias voltage dependence of heat flow, this result exceeds the +10% heat flow switching ratio observed in the first device. Despite the higher lattice thermal conductivity of the electrode material compared to that in the first device, this improvement is primarily attributed to the substantial reduction in the electrode thickness, which leads to an increase in the proportion of volume where carrier concentration changes occur. Importantly, as seen in the raw TDTR data in Figure 3(b), differences were also observed in the relaxation curves over longer time scales (>30 ns), indicating that the total heat flow through the device is also switched.

The plausible explanation for the decreased heat flow when applying a negative bias voltage is the formation of a depletion layer at the interface between the Mo layer and the Si-Ge-Au electrode layer. While this effect might be difficult to detect using the AC heating method shown in Figure 2(a), the TDTR method directly measures the surface temperature response after pulse heating, making it possible to detect the influence of such localized phenomena.

Figure 3(b) also shows the bias voltage dependence of leakage current observed in the second heat flow switching device. When the bias exceeded approximately *V*_B_ = +50 V, leakage current through the amorphous SiO_2_ dielectric layer increased, causing the suppression of linear heat flow variation to bias voltage. To improve the heat flow switching ratio, we need to establish techniques for fabricating high-quality thin films of insulating materials with relatively high dielectric constants.

### Thin film silver chalcogenide electrodes and ionic liquid dielectric layer

To address the limitations of the second device and further enhance heat flow switching ratio, we developed a third device utilizing a thin-film silver chalcogenides electrode and an electric double-layer capacitor (EDLC) structure with an ionic liquid dielectric [34].

**Figure 4.**
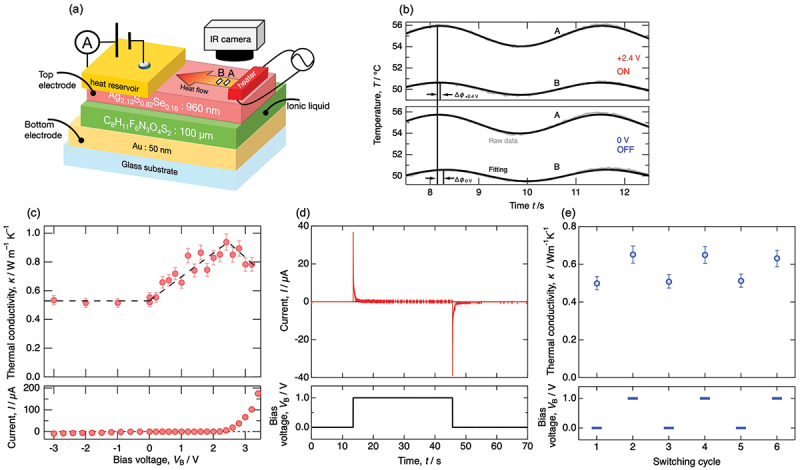
(a) Schematic illustration of electric double-layer capacitor-type heat flow switching device and bias voltage-dependent thermal conductivity measurement setup using an IR camera. (b) Time-dependent temperature wave measured at the points of A and B, separated by 0.15 mm on the surface of silver chalcogenides electrode, with *V*_B_ = +2.4 V and 0 V. (c) Bias voltage dependent thermal conductivity and leakage current. (d) Transient current response during charging and discharging process at *V*_B_ = +1 V. (e) Repeated thermal conductivity measurements under alternating application of *V*_B_ = 0 V and 1 V (without leakage current) for three cycles. These figures are reconstructed from the data of Ref. [34].

As shown in Figure 4(a), the third-generation device primarily consisted of: an Ag_2_S_0.8_Se_0.2_ electrode with exceptionally low lattice thermal conductivity (~0.5 W m^−1^ K^−1^), an ionic liquid (1-ethyl-3-methylimidazolium bis(trifluoromethanesulfonyl)imide) dielectric layer with high dielectric constant, and an Au thin film counter electrode. This EDLC-type heat flow switching device, capable of achieving extremely high capacitance density, was expected to provide improved heat flow switching ratio and reduced operating voltage compared to conventional devices due to its enhanced charge storage capability. The Ag_2_S_0.8_Se_0.2_ layer (960 nm in thickness) was deposited on a porous cellulose membrane using molecular beam deposition (MBD). Ideally, the electrode thickness should be approximately a few nanometers to maximize the proportion of the layer contributing to electron thermal conductivity changes. However, the porous cellulose membrane used as a substrate had a root mean square roughness of 117 nm and a maximum height difference of 721 nm. Consequently, an Ag_2_S_1–*x*_Se_*x*_ film with a thickness of 960 nm was deposited to ensure a flat surface for measurement. The bottom Au electrode was formed on a glass substrate by RF magnetron sputtering to a thickness of 50 nm. The device was assembled by stacking three components: (glass substrate + Au electrode) / (ionic liquid-impregnated cellulose separator) / (Ag_2_S_0.8_Se_0.2_ electrode).

Silver paste and copper wire were used to connect the electrodes to the external measurement system. The bias voltage dependence of thermal conductivity was measured using a periodic heating method. The time-dependent temperature wave was measured at two points (A and B) on the silver chalcogenide electrode surface, which were 0.15 mm apart. An electrometer was also connected to the bias voltage application circuit to simultaneously measure instantaneous current and leakage current during bias voltage application.

Figure 4(b) shows the raw data of the time-dependent temperature wave used for the following thermal conductivity estimation at a bias voltage of *V*_B_ = +2.4 V and zero bias voltage. A smaller phase difference of temperature wave means faster heat propagation, so that the applied bias voltage causes a significant increase in thermal conductivity. Figure 4(c) shows the bias voltage dependence of thermal conductivity and leakage current observed in the device. The data showed further improvement in heat flow switching performance compared to the first and second devices. Thermal conductivity increased linearly with increasing bias voltage, achieving a maximum switching ratio of 1.9 at *V*_B_ = +2.4 V compared to the zero-bias voltage. This represents approximately twice the performance improvement compared to the first device, which showed +10% switching ratio by changing *V*_B_ from 0 V to +3 V. Furthermore, during repeated switching tests, variations in thermal conductivity were observed with good reproducibility and no significant performance degradation, suggesting high stability of the electrode-electrolyte interface and absence of irreversible electrochemical reactions. When the bias voltage exceeded +2.4 V, thermal conductivity began to decrease. This is most likely due to the inability to accumulate carriers as evidenced by a significant increase in leakage current.

The response time and accumulated charge of this EDLC-type heat flow switching device were also evaluated by measuring the instantaneous current during bias application and removal. As shown in Figure 4(d), when applying *V*_B_ = +1 V, an initial current spike was observed, rapidly decaying to nearly zero within 0.18 seconds. This behavior is consistent with the typical charging behavior of capacitors [35], indicating carrier accumulation at the electrode-electrolyte interface on a sub-second timescale. The accumulated charge calculated from the discharge current after bias removal was *Q* = 9.89 × 10^−6^ C. Using an electrode area *S *~ 0.5 cm^2^ and a roughly estimated accumulation layer thickness *t* = 1 nm, the carrier concentration change was estimated to be Δ*n *~ 1.2 × 10^21^ cm^−3^. Consequently, applying the Drude model and Wiedemann-Franz law, an increase in electron thermal conductivity is expected to be ~0.7 Wm^−1^K^−1^, showing good agreement with the experimental results.

Figure 4(e) shows the cyclic characteristics demonstrating the reversible change in thermal conductivity of the capacitor-type heat flow switching device. The thermal conductivity was measured while alternately applying *V*_B_ = 0 V and 1 V (without leakage current) for three consecutive cycles. The results clearly indicate that the thermal conductivity changes reversibly with the applied bias voltage, and no noticeable degradation in switching performance is observed during repeated operation.

## Discussion

The evolution of capacitor-type heat flow switching devices across three generations represents a systematic progression in materials selection, device architecture, and performance optimization. Table 1 summarizes the key characteristics and performance metrics of each generation. Several key trends and insights emerge from this progression. Electrode thickness optimization demonstrated that reducing electrode thickness from 10 μm to 40 nm enhanced switching performance by increasing the proportion of material experiencing carrier concentration modulation. All three generations employed materials with exceptionally low lattice thermal conductivity (0.4 – 0.7 W m^−1^K^−1^), confirming the critical importance of minimizing the lattice contribution. Dielectric innovation showed that the transition from solid dielectrics (amorphous Si, SiO_2_) to an ionic liquid in the third-generation device enabled higher capacitance density and lower operating voltage while reducing leakage current issues. Operating voltage optimization revealed that the operating voltage for maximum switching ratio varied significantly across generations (*V*_B_ = +3 V → +50 V → +2.4 V), with the third-generation device achieving the best performance at the lowest voltage due to the electric double-layer capacitor structure. Response dynamics analysis showed that the third-generation device demonstrated rapid response (0.18 s), confirming the inherently fast dynamics of carrier accumulation mechanisms compared to alternative switching approaches based on phase transitions or mechanical actuation.

**Table 1.** Key characteristics and performance of the three-generation capacitor-type heat flow switching devices developed in our research.

	First generation	Second generation	Third generation
Electrode material	Ag_2_S_0.6_Se_0.4_	Si_37.5_Ge_57.5_Au_5_	Ag_2_S_0.8_Se_0.2_
Electrode thickness	10 μm	40 nm	960 nm
Dielectric layer	Amorphous Si	SiO_2_	Ionic liquid
Heat flow direction to the carrier accumulated layer	In-plane	Cross-plane	In-plane
Maximum switching ratio	1.1	1.55	1.9
Required bias voltage	+3 V	+50 V	+2.4 V
Response time	—	—	0.18 s
Lattice thermal conductivity	0.4 W m^−1^K^−1^	0.7 W m^−1^K^−1^	0.4 W m^−1^K^−1^
Electrical conductivity	~10 S cm^−1^	~8 S cm^−1^	~10 S cm^−1^

The capacitor-type heat flow switching devices offer several distinctive advantages. For example, the temperature-independent operation principle distinguishes our devices from phase-change materials and VO_2_-based thermal switches [3]. These devices operate over a wide temperature range without relying on specific transition temperatures, enabling more versatile thermal-management solutions. Although the maximum switching ratio of our device (1.9) is comparable to that of VO_2_-based thermal switches [3], its rapid response time of 0.18 s demonstrates that the third-generation device exhibits one of the fastest response characteristics among non-mechanical switching technologies, making it suitable for dynamic thermal-management applications requiring quick adjustments. In contrast to VO_2_-based or other phase-change materials, where repeated structural transitions can induce volume changes and lead to performance degradation, our capacitor-type devices exhibit good reproducibility because their operation relies solely on carrier modulation without structural transition. Solid-state design emphasizes that the absence of moving parts, fluids, or significant phase changes enhances reliability and enables miniaturization for integration with microelectronic systems. Low operating voltage highlights that the third-generation device operates at just *V*_B_ = +2.4 V, making it compatible with standard electronic control systems without requiring specialized high-voltage power supplies. However, due to the limitations of ionic liquids in terms of temperature range and long-term stability, the exploration of highly stable ionic liquids or their replacement with solid electrolytes remains a challenge for future work.

The primary limitation of our current devices is the small heat flow switching ratio of 1.9 times [34], which remains lower than some alternative technologies such as phase change materials, electrochemical methods or mechanical switches. However, as discussed below, several promising pathways exist for further enhancing this performance metric without sacrificing the inherent advantages of the capacitor-type approach.

**Figure 5.**
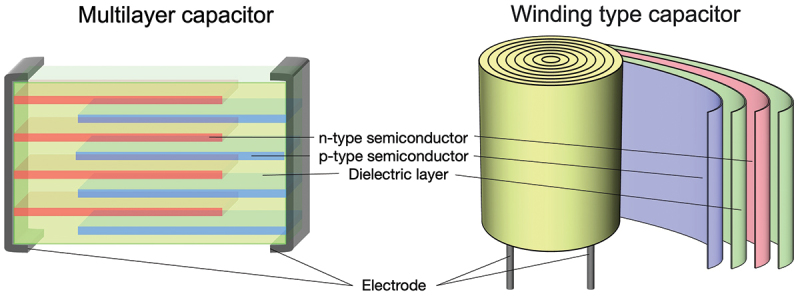
Schematic illustration of multilayer and winding-type structures contributing to the enhancement of the heat flow switching ratio in capacitor-type heat flow switching devices.

While significant progress has been made, several challenges and opportunities remain for further advancement of capacitor-type heat flow switching technology. Exploring materials with ultra-low lattice thermal conductivity could substantially enhance switching ratios by investigating materials with *κ*_lat_ below 0.4 W m^−1^K^−1^. Promising candidates include Ag_5–*x*_Te_3_ (*κ*_lat_ ~ 0.25 W m^−1^K^−1^ [36]), which possesses strong anharmonic lattice vibrations most likely due to its ionic conduction nature, and other low lattice thermal conductivity semiconductors, such as WSe_2_, AgSbTe_2_, etc [37–39]. In addition, as shown in Figure 5, multilayer device structures could significantly increase the proportion of material experiencing modulation since carrier accumulation occurs primarily near electrode-dielectric interfaces. By utilizing the ductility and flexibility of silver chalcogenide-based materials [27–29], it would also be possible to fabricate heat flow switching devices with a cylindrical structure of a wound capacitor. Preliminary estimation suggests that adopting multilayer structures with each layer being a few tens of nanometers in thickness could potentially achieve switching ratios exceeding 10 times [20].

To achieve a high thermal switching ratio, it is essential not only to minimize the lattice thermal conductivity (*κ*_lat_) but also to carefully select electrode materials that maximize the relative change in electronic thermal conductivity, Δ*κ*_ele_ / *κ*_ele_(0), which corresponds to Δ*σ* / *σ*(0) according to the Wiedemann-Franz law. The magnitude of this change is strongly governed by the intrinsic electronic properties of the electrode, particularly the initial carrier concentration and the band gap. Materials with a relatively low initial carrier concentration (*n*_0_) allow a larger modulation of carrier density (Δ*n* / *n*_0_) through the field effect; however, if *n*_0_ is too low, the material becomes insulating, making carrier injection difficult. The band gap also plays an important role, as it determines both the intrinsic carrier concentration and influences the attainable range of carrier modulation by the field. A moderate band gap is desirable: a smaller band gap leads to metallic behavior with limited carrier modulation, whereas a larger band gap increases the barrier for carrier injection and suppresses field-effect control. Consequently, we consider that the electrode material should possess semiconducting characteristics with a moderate initial carrier concentration (*n*_0_ = 10^18^–10^20^ cm^−3^) and a band gap (*E*_g_ = 0.1–1 eV) in addition to ultralow lattice thermal conductivity (≤0.5 W m^−1^ K^−1^).

Advanced dielectric engineering with novel ionic liquids featuring wider electrochemical windows (>5 V) and higher ionic conductivity could enable higher bias voltages without leakage current limitations. In addition, to further improve the durability and practical applicability of the device, it is also important to replace the ionic liquid in the dielectric layer with solid dielectrics possessing high dielectric constants (e.g. HfO_2_, ZrO_2_ [40]) while maintaining a high breakdown voltage.

In our study, we confirmed that clear heat-flow switching was observed in both the in-plane (Figures 2(a) and 5(a)) and cross-plane (Figure 3(a)) configurations. The amount of carrier accumulation at the interface does not depend on the measurement configuration (i.e. the direction of heat flow). However, the observable change in heat flow caused by the accumulated carrier layer – where the thermal conductivity is modulated – could vary depending on the measurement configuration. For example, considering the relative arrangement of the carrier-accumulated layer and the layer that does not contribute to the modulation of thermal conductivity within the electrode, the in-plane configuration allows these two layers to be arranged in parallel. As a result, the carrier-accumulated layer, which exhibits higher thermal conductivity, can be effectively utilized as the main heat-conduction path in the electrode. In contrast, in the cross-plane configuration, the two layers are arranged in series in the electrode. Because electrode materials typically possess low lattice thermal conductivity, the non-modulating layer can act as a substantial thermal resistance, thereby suppressing the overall modulation. Consequently, the in-plane configuration is expected to be more suitable for achieving a larger thermal switching ratio.

The difference in heat-flow direction between the two configurations also affects the selection of dielectric materials for future performance improvement. For the in-plane configuration, the dielectric layer should have low thermal conductivity so that the heat flow predominantly passes through the electrode region, where the thermal conductivity modulation occurs. In contrast, for the cross-plane configuration, the dielectric material should have high thermal conductivity to ensure that the thermal-conductivity modulation in the electrode is not masked.

Practical aspects should also be considered. Although the two configurations operate on the same basic principle, the cross-plane configuration is more suitable for compact electronic devices, where space is severely limited, as it enables vertical stacking directly beneath the target component.

The unique features of capacitor-type heat flow switching devices make them particularly well-suited for several emerging application areas [3,4]. In thermal management for electronics, active control of heat dissipation in high-performance computing systems enables dynamic adjustment of cooling strategies based on processing loads and ambient conditions. For energy harvesting optimization, these devices can enhance the efficiency of thermoelectric generators by dynamically controlling thermal gradients to match changing environmental conditions. In thermal computing and logic applications, the implementation of thermal analogues to electronic components for information processing using heat instead of electricity potentially offers energy advantages for specific computational tasks. For spacecraft thermal control, precise management of heat distribution in satellite and space vehicle systems becomes possible, where traditional convective cooling is unavailable, and reliability requirements are extreme. In innovative building materials, integration into construction elements enables active control of thermal energy flow for improved energy efficiency in heating and cooling applications.

## Summary

Our research has demonstrated the development and progressive improvement of capacitor-type heat flow switching devices based on modulation of electron thermal conductivity through carrier concentration control. The key achievements include a proof-of-principle demonstration, which established the viability of controlling heat flow through bias voltage-induced carrier concentration modulation, with initial devices showing a 10% increase in heat flow at *V*_B_ = +3 V. Materials engineering identified and characterized silver chalcogenide and amorphous Si-Ge-Au materials with exceptionally low lattice thermal conductivity (0.4 – 0.7 W m^−1^ K^−1^) combined with appropriate semiconductor transport properties, enabling effective thermal conductivity modulation. Device architecture innovation progressed from bulk electrodes to thin-film structures and ultimately to electric double-layer configurations, dramatically improving performance at each stage. Performance enhancement increased the maximum switching ratio from 1.1 to 1.9 while slightly reducing the operating voltage from *V*_B_ = +3 V to *V*_B_ = +2.4 V in the most advanced device. Response characterization demonstrated rapid switching response (0.18 s) and excellent cyclability, confirming the practical viability of the device for dynamic thermal management applications. These accomplishments establish capacitor-type heat flow switching as a promising technology platform offering unique advantages in operation principle not restricted by a phase-transition temperature, response time, and integration potential compared to alternative approaches.

## Data Availability

The data that support the findings of this study are available from the corresponding author upon reasonable request.
